# *OsSGL*, a novel pleiotropic stress-related gene enhances grain length and yield in rice

**DOI:** 10.1038/srep38157

**Published:** 2016-12-05

**Authors:** Manling Wang, Xuedan Lu, Guoyun Xu, Xuming Yin, Yanchun Cui, Lifang Huang, Pedro S. C. F. Rocha, Xinjie Xia

**Affiliations:** 1 Key Laboratory of Agro-ecological Processes in Subtropical Region, Institute of Subtropical Agriculture, Chinese Academy of Sciences, Changsha, Hunan 410125, China

## Abstract

Abiotic stress seriously affects the yield of rice (*Oryza sativa* L.). Grain yield in rice is multiplicatively determined by the number of panicles, number of grains per panicle, and grain weight. Here, we describe the molecular and functional characterization of *STRESS_tolerance and GRAIN_LENGTH (OsSGL*), a rice gene strongly up-regulated by a wide spectrum of abiotic stresses. *OsSGL* encodes a putative member of the DUF1645 protein family of unknown function. Overexpression of *OsSGL* significantly altered certain development processes greatly and positively affecting an array of traits in transgenic rice plants, including increased grain length, grain weight and grain number per panicle, resulting in a significant increase in yield. Microscopical analysis showed that the enhanced *OsSGL* expression promoted cell division and grain filling. Microarray and quantitative real-time PCR (qRT-PCR) analyses revealed that a large number of genes involved in stress-response, cell cycle and cytokinin signaling processes were induced or suppressed in *OsSGL-*overexpressing plants. Together, our results suggest that *OsSGL* may regulate stress-tolerance and cell growth by acting via a cytokinin signaling pathway. This study not only contributes to our understanding of the underlying mechanism regulating rice stress-tolerance and grain length, but also provides a strategy for tailor-made crop yield improvement.

A doubling of rice production per hectare will be necessary to meet projected requirements of this crop by 2050^1^,^2^,^3^. Worldwide, abiotic stresses such as drought, salinity and high/low temperatures cause substantial (>50%) losses in yield annually^4^. Thus, two major challenges, improving of stress-tolerance and increasing crop yield, have been the key focuses of rice breeding programs with significant efforts having been made to dissect the physiology, genetic, and molecular biology bases of the two complex traits^5^,^6^.

It is known that a substantial network of regulatory interactions and coordination of distinct pathways underlies plant responses to different abiotic stresses^5^,^6^,^7^,^8^. Numerous genes have been identified that participate in regulating plant stress-tolerance at various developmental processes and stages in different pathways through diverse mechanisms^9^,^10^. Plant genetic modification by insertion of genes involved in stress response pathways is one approach to increase stress-tolerance in crops. However, transgenically improved rice with enhanced stress-tolerance may suffer detrimental physiological and morphological effects during normal growth conditions, such as reduced plant growth, which may result in significant reduction of potential yield^8^,^10^,^11^. Rice yield, which is multiplicatively determined by the number of panicles per plant, number of grains per panicle and grain weight, is controlled by non-allelic genes and quantitative trait loci (QTLs)^1^,^12^,^13^,^14^,^15^. More than 400 QTLs associated with rice grain traits have been identified, but no more than 40 of these have been cloned or genetically fine-mapped^12^,^13^. Thus, most yield trait genes and their molecular mechanisms remain to be cloned and elucidated. In addition, some genes (*e.g. OsTB1*^16^, *DTH8*^17^, *GS3*^18^, *GW2*^19^, *qSW5*^20^ and *TGW6*^21^) are negative regulators of grain yield, and the number of known genes applicable for simultaneously improving both stress-tolerance and yield is very limited.

## Results

### *OsSGL* is Highly Induced by Multiple Stresses

To facilitate the analysis and molecular characterization of the regulatory network of stress-related genes in rice, we carried out a microarray-based study of global genome expression profiles of a male-sterile *indica* cultivar, Pei’ai 64 S (PA64S), subjected to cold, heat or drought stresses using Affymetrix GeneChip^®^ arrays^22^,^23^,^24^,^25^. The analysis of microarray data showed that many genes were up- and down-regulated by different stresses in the rice genome (unpublished data). Thirty of the genes were highly (>4-fold) responsive to all three stresses used in both tissues (leaf and panicle) of rice at all three developmental stages (seedling, booting and heading). *OsSGL* was one of the genes significantly up-regulated with up to 33.86-fold increases in leaves and panicles at both seedling and booting stages, especially in cold-treated samples (Supplementary Table S1). Search of the Gene Expression Omnibus repository at NCBI showed that *OsSGL* is also highly induced by exposure to blast fungus, sodium arsenate, benzothiadiazole and salinity stress-treatments.

Further analysis of the temporal and spatial expression of *OsSGL* by qRT-PCR showed that the gene was ubiquitously expressed at 1.13 to 43.57-fold higher levels in stressed plants compared with control plants, with the highest expression being observed in cold stressed leaves at booting stage (Supplementary Table S1). The differential expression patterns corresponded well with the microarray data obtained and confirmed that *OsSGL* was strongly induced by a wide spectrum of stresses in different tissues at different developmental stages of rice, suggesting that *OsSGL* may participate in stress resistance.

### Characterization of *OsSGL*

By referencing the microarray probe information, *OsSGL* is located on chromosome 2, and a corresponding cDNA containing its entire ORF was cloned from PA64S (NCBI accession AK108331.1). The full-length cDNA is GC-rich (68.7% GC) and includes 105 bp of 5′ UTR, 768 bp of coding region and 217 bp of 3′ UTR. Alignment of the cDNA with the genome sequence of Nipponbare (ssp. *japonica*) showed that *OsSGL* contains a single exon encoding a putative 255-aa protein of unknown function belonging to the DUF (Domains of Unknown Function) 1645 protein family (Supplementary Fig. S1). No polymorphisms were detected in the ~3 kb sequence region including the entire *OsSGL* ORF and a 2-kb fragment 5′ upstream of it between Nipponbare and 93-11 (ssp. *indica*). Alignment of the nucleotide and corresponding polypeptide sequences of *OsSGL* with homologous ones of other species using CLUSTALW showed a high DNA similarity to *O. rufipogon* (98%, NCBI accession CU406729.1), but much lower DNA similarities to other species (up to 31% to the sorghum (*Sorghum bicolor*); NCBI accession XM_002453222.1), whereas the polypeptide similarities to non-*Oryza* species were up to 53.3% with maize (*Zea mays;* NCBI accession NP_001145342.1). No known-function domains and transmembrane regions were detected in the predicted OsSGL sequence by CD-Search, TMHMM and TMpred analysis.

To elucidate the cellular localization of OsSGL, a reporter fusion gene of *OsSGL* to *GFP* was introduced into onion epidermis cells. Compared with the CaMV35S::GFP control, where GFP signal was distributed on the plasma membrane, cytoplasm and nucleus, the OsSGL*-*GFP fusion protein was detected only in the nucleus (Supplementary Fig. S2). However, no nuclear targeting signals were predicted in OsSGL by SignalP, ProtComp and WoLFPSORT analyses.

To assess the spatial and temporal expression pattern comprehensively, the tissue specificity of *OsSGL* expression was investigated in the transgenic rice harboring an *OsSGL* promoter::GUS gene fusion construct. GUS activity was observed in leaf, leaf sheath, node, internode, young coleoptile, root, palea and lemma of young florets, and stamens and pistils of young florets before flowering (Supplementary Fig. S3A–H). Both in transverse and longitudinal sections of roots, GUS activity was mainly detected in the vascular bundle of the pericycle, especially in the lateral root apical meristem where cells actively proliferate (Supplementary Fig. S3I). Furthermore, GUS activity was also highly detected in the parenchyma and phloem tissue of leaves as seen in transverse sections (Supplementary Fig. S3J). Such observations were consistent with our microarray results and the RiceXPro expression data. The high levels of expression in these tissues suggest that *OsSGL* may play an important role in regulating rice vegetative and reproductive developments.

### *OsSGL* Enhances Rice Yield Significantly

The effects of *OsSGL* on plant development and stress resistance were analyzed in transgenic plants harboring the *OsSGL*-overexpressing construct (OE). Four *indica* rice cultivars, 93-11, Huinong10 (HN10), Minghui 63 (MH63) and KH2, and a *japonica* rice cultivar, Taibei 309 (TB309), were used in the transformation of plants with OE. For 93-11, a total of 23 transgenic plants (T_0_) were obtained as detected by both PCR and Southern blot analysis (data not shown). The expression levels of *OsSGL* were examined in all 23 lines by qRT-PCR (Supplementary Fig. S3). *OsSGL* transcripts were significantly more abundant in the transgenic lines relative to the control plant with large differences among them.

**Figure 1:**
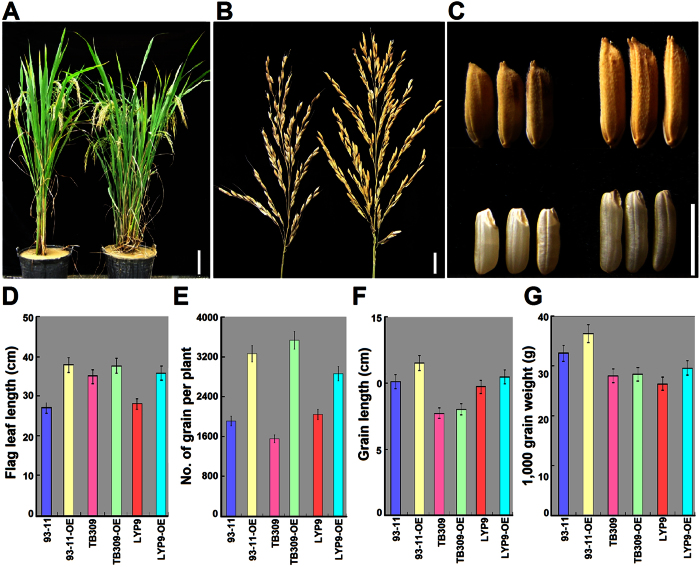
Comparisons of agronomic traits of mature plants, panicles, grains and flag leaf between wild-type rice 93-11 (WT) and *OsSGL*-overexpression 93-11-OE transgenic lines. (**A–C**) Gross morphology of 93-11 (left) and 93-11-OE (right) mature plants (**A**), panicles (**B**) and grains (**C**). Scale bars, 20 cm, 1 cm and 1 cm, respectively. (**D–G**) Comparisons of grain length (**D**), grain number per plant (**E**), weight of 1,000 grains (**F**) and grain weight per plant (**G**). Values are measured as means with SD (n = 10 plants). 93-11, *indica* WT; 93-11-OE, *indica* transgenic lines; TB309, *japonica* WT; TB309-OE, *japonica OsSGL*-overexpressing transgenic lines; LYP9, PA64S/93-11 heterozygote; LYP9-OE, PA64S/93-11-OE transgenic heterozygote.

Phenotype analysis showed that all transgenic lines exhibited a broadly normal phenotype at the vegetative growth stage. Remarkably, during plant reproductive development, 93-11-OE plants exhibited alterations in a number of agronomic traits such as plant height, panicle architecture, grain length and grain weigh in field conditions (Fig. 1A–G and Supplementary Table S3). Compared with the wild-type 93-11 (WT), transgenic 93-11-OE plants showed slightly lower stature (Fig. 1A) but enlarged panicles (Fig. 1B). The 93-11-OE panicle produced more primary and secondary branches, led to a 22.2% increase in panicle length with an average increase of 25.7% in grain number per panicle than WT (Fig. 1B and E and Supplementary Table S3). The grains of 93-11-OE were on average 24.8% longer, 8.6% narrower and 16.3% heavier (Fig. 1C and D–G and Supplementary Table S3) than those of WT, theoretically leading to an average 46.2% increase in grain yield per panicle. Broadly similar phenotypic changes were also observed with the other four OE transgenic varieties obtained (Supplementary Table S3 and Fig. S5). To explore the application of 93-11-OE lines in hybrid rice breeding, we crossed transgenic line C3–1 with PA64S, a commercial photothermosensitive male sterile line, to generate F1 hybrid (heterozygote) seeds. Compared with hybrid LYP9 (Liangyoupei 9) derived from the cross of PA64S and 93-11 (WT), the PA64S/93-11-OE hybrid (LYP9-OE) produced significantly longer flag leaves and more, longer and heavier grains, resulting in an average increase of 12.1% in grain yield in field trials (Fig. 1D–G).

Transgenic plants harboring the *OsSGL-*suppression construct (RNAi) were also analyzed in 93-11 and KH2, and the expression levels of *OsSGL* were examined in all 93-11-Ri T_0_ lines by qRT-PCR (Supplementary Fig. S6). Compared to WT, the relative lower expression levels (0.02–0.33 of WT levels) were detected in *OsSGL*-RNAi transgenic plants. However, the phenotypic examination showed no obvious differences between WT and *OsSGL*-RNAi transgenic plants during the growth and developmental stages.

### Biological Role of *OsSGL*

**Figure 2:**
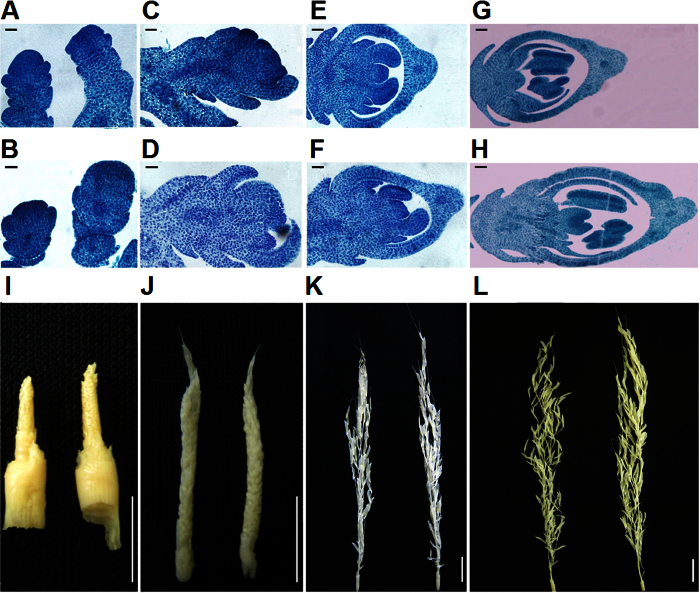
Comparison of rice panicles at different development stages between 93-11 and 93-11-OE. (**A** and **B**) Microscope images of longitudinal sections of the shoot apical meristem of 93-11 (**A**) and 93-11-OE (**B**) at the transition stage from the vegetative to the reproductive phase. Scale bar, 20 μm. (**C** and **D**) Microscope images of longitudinal sections of 93-11 (**C**) and 93-11-OE (**D**) spikelets at the primary branches formation stage. Scale bar, 20 μm. (*E* and *F*) Microscope images of longitudinal sections of 93-11 (**E**) and 93-11-OE (**F**) spikelets at the secondary branches formation stage. Scale bar, 20 μm. (**G** and **H**) Microscope images of longitudinal sections of 93-11 (**G**) and 93-11-OE (**H**) spikelets at the flower organs differentiation stage. Scale bar, 200 μm. (**I–L**) Panicles of 93-11 (left) and 93-11-OE (right) of 1 cm (*I*), 3 cm (*J*), 10 cm (*K*) and 20 cm (**L**). Scale bar, 1 cm and 3 cm, respectively.

To elucidate the possible function of *OsSGL,* we analyzed the effects of its overexpression on spikelet and panicle developments. Microscopical examination of the developmental processes of spikelets and panicles of 93-11 and 93-11-OE plants grown in parallel showed that the rachis meristem and spikelets at both primary and secondary branch primordia formation and flower organ differentiation stages were markedly larger in 93-11-OE than those observed in the wild-type 93-11 (Fig. 2A–H). However, no obvious difference was observed in panicle length at the time when panicles reached about 1–3 cm long between wild-type and 93-11-OE panicles (Fig. 2I and J). Nevertheless, the panicle of 93-11-OE was significantly longer than that of 93-11 (Fig. 2K–L) at the time when the panicle reached 10 cm long and over. Therefore, the phenotype of longer panicles in 93-11-OE appeared at the late stage of panicle development.

**Figure 3:**
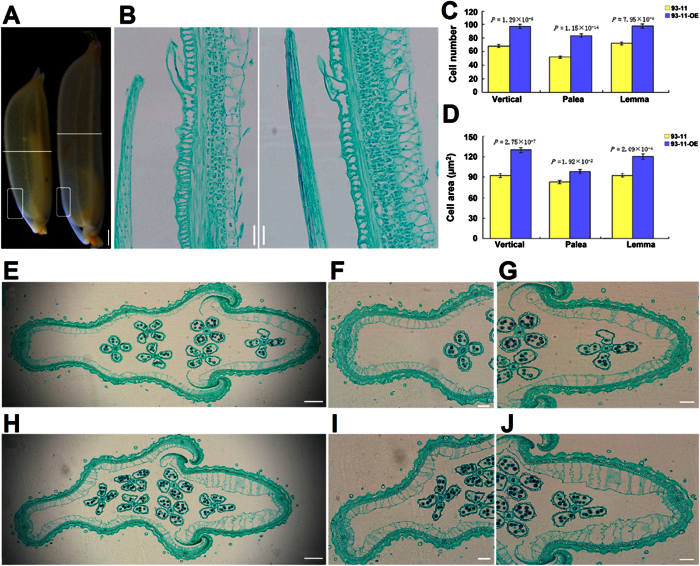
The effect of *OsSGL* on cell number and size in lemma/palea. (**A**) Spikelets of 93-11 (left) and 93-11-OE (right) 6 days before heading. Scale bar, 2.5 mm. (**B**) Magnifications of indicated squares cut at the middle of the longitudinal axis of the panicle. Scale bar, 20 μm. (**C** and **D**) Comparisons of parenchyma cell numbers (**C**) and sizes (**D**) between 93-11 and 93-11-OE in the longitudinal sections and cross-sections of the inner parenchyma cell layer of spikelets. (**E–J**) Cross-sections of florets cut horizontally as shown in *A*. (**F** and **I**) lemma; (*G* and *J*) palea. 93-11, the middle panels (**E–G**); 93-11-OE, the bottom panels (**H–J**). Scale bar, 200 μm and 100 μm, respectively. All *P* values are based on two-tailed *t*-tests. Yellow bars, 93-11; blue bars, 93-11-OE. Values are measured as means ± s.e.m. (n = 12 plants).

The effect of overexpression of *OsSGL* on cell number and size was investigated in lemma/palea of rice plants. Transverse and longitudinal sections of the central parts of the palea/lemma of florets were microscopically examined before fertilization and compared between 93-11 and 93-11-OE, which produced much longer spikelets than 93-11 (Fig. 3A–J). Observation of a cross section of the spikelets revealed that the inner parenchyma cell layer of palea/lemma in 93-11-OE contained 35.0–60.5% more cells than in the 93-11 hull and that its cells were 18.4–29.6% larger (Fig. 3C–J). Furthermore, inspection of longitudinal palea and lemma sections showed that the inner parenchyma cell layer of 93-11-OE contained 42.7% more cells than 93-11, which were on average 40.3% larger than wild-type cells (Fig. 3B–D). These results demonstrate that *OsSGL* positively affects grain size by increasing both cell number and cell size leading to the enhanced longitudinal growth of the rice grains. The positive regulation of grain size by *OsSGL* contrasts with findings for other known genes, which are negative regulators of rice grain size, such as *OsTB1*^16^, *DTH8*^17^, *GS3*^18^, *GW2*^19^, *qSW5*^20^ and *TGW6*^21^.

Comparison of the grain-filling rates between 93-11 and 93-11-OE revealed an increased grain-filling rate in 93-11-OE (Supplementary Fig. S7). Both the fresh weight (FW) and dry weight (DW) of 93-11-OE grains were lower than those of the wild-type up to 5 days after fertilization (daf). However, starting at 5 daf, both FW and DW of transgenic lines increased significantly faster, and at 17 daf the FW and DW of 93-11-OE grains were 33.4% and 28.1% heavier than those of 93-11 grains, consistent with the longer ovaries and grains observed in 93-11-OE (Supplementary Fig. S8). Therefore, *OsSGL* might also play a role in dry matter accumulation during grain milk filling, thereby regulating grain weight.

Unexpectedly, a coil at the base of the flag leaf was exclusively present in all OE-transgenic plants (Supplementary Fig. S9). The flag leaf of OE-transgenic plants was longer but slightly narrower, and the leaf area was larger (average 21.9% in 93-11 and 8.1% in TB309) than that of controls (Supplementary Table S3). This interesting morphological trait decreased the lamina joint angle between the flag leaf and the culm in the transgenic plants. The effect of the leaf rolling phenotype on other important production traits and stress-tolerance in rice is currently under investigation.

Under normal growth conditions, seedlings of transgenic plants grew faster and exhibited longer roots and shoots (9.4 ± 1.1 cm and 16.9 ± 1.6 cm, respectively) than WT (7.3 ± 0.9 cm and 12.6 ± 1.1 cm, respectively; Supplementary Fig. S10). To investigate a possible role of *OsSGL* in drought resistance, transgenic 93-11-OE and 93-11 wild-type control plants were submitted to moderate drought stress with 20% (M/V) PEG6000 in hydroponics, a condition that is not lethal to wild type plants. In the moderate drought treatment, all transgenic plants lost water more slowly from leaves and consequentially showed much delayed leaf-rolling compared with wild-type control plants. When nearly all leaves of wild-type plants had completely rolled, only a small fraction of the leaves of the transgenic plants had slightly rolled. After the 7 day-long moderate stress treatment, the lengths of both roots and shoots of the transgenic lines were significantly (*p*<0.01) longer than those of the wild-type following the stress and recovery phases. The average lengths of roots and shoots of transgenic plants were 52.3 ± 2.6 mm and 33.4 ± 1.3 mm respectively in transgenic plants, but only 33.5 ± 2.7 mm and 20.4 ± 1.0 mm long, respectively, in the control plants (Supplementary Fig. S11). Moreover, our newly obtained data also showed that overexpression of *OsSGL* enhanced drought tolerance of the transgenic lines and promoted plant growth with and without exposure to different levels of drought stress (view detail in a separate manuscript).

Thus, overexpression of *OsSGL* greatly and positively affected rice growth and productivity with the transgenic plants producing longer grains and larger panicles with more grains per panicle, along with an atypically coiled flag leaf, consequently resulting in a marked improvement of grain yield. In addition, the morphological marker of curling flag leaves produced together with the long-grain phenotype greatly facilitated the selection of positive transgenic plants and the downstream breeding.

### *OsSGL* May Function via Cytokinin Signal Transduction Pathway

**Figure 4:**
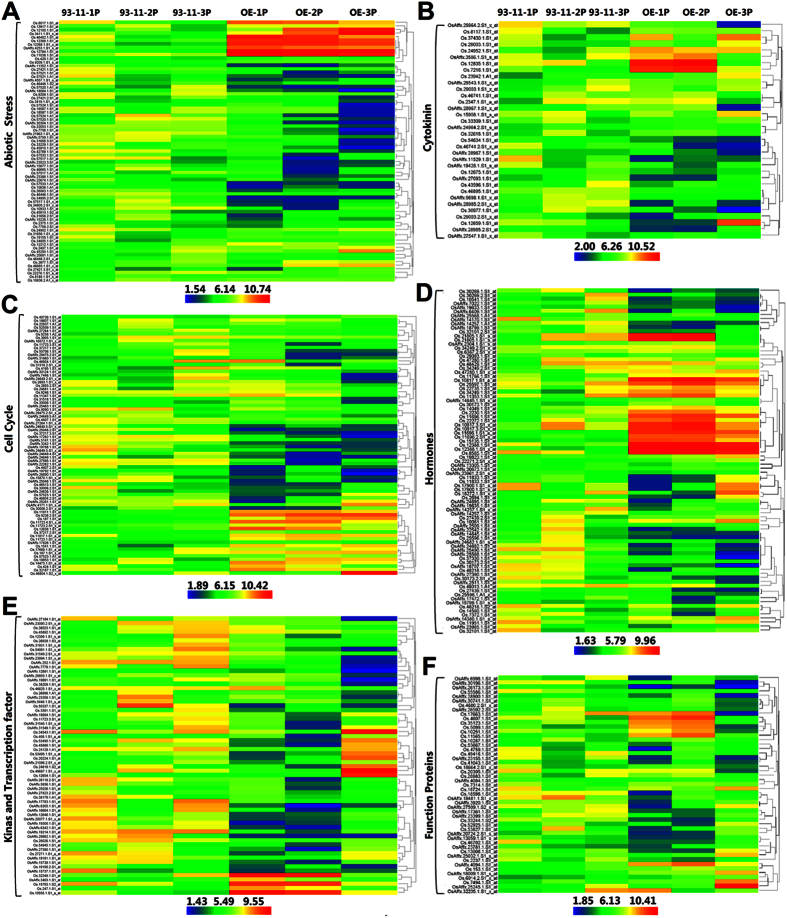
Hierarchical cluster analysis of the significantly impacted genes in 93-11-OE at the floral development stage under normal conditions. Heatmaps represent mean expression values (n = 3) for *OsSGL*-overexpressing line C3–1 and wild-type 93-11. Genes impacted by *OsSGL* overexpression as revealed by microarray analysis include genes involved in (**A**) abiotic stress response (73 genes), (**B**) cytokinin receptor/signaling (34 genes), (**C**) cell cycle (76 genes), (**D**) plant hormones (GA, BR, IAA, ethylene and ABA) response/biosynthesis/signaling (119 genes), (**E**) kinase and transcription factors (64 genes) and (**F**) proteins functioning in metabolic processes (49 genes). 1 P, young panicle of 3 to 6 cm in length before heading; 2 P, panicle over 15 cm 3 days before heading; 3 P, panicle 10 days after heading. The signal values are log_2_-transformed and subjected to complete linkage hierarchical clustering with HemI program^68^. The colored bars at the bottom represent the z-score values transformed from log_2_-based expression values with blue color indicating low level (down-regulation), green indicating medium level, and red signifying high level (up-regulation).

To test the hypothesis that *OsSGL* enhances meristematic activity and promotes cell proliferation, an initial microarray analysis of panicles of wild-type 93-11 and 93-11-OE at different developmental stage was performed to identify genes impacted by *OsSGL*. In total, 3939 significantly differentially expressed genes (DEGs) were determined by more than 2-fold expression change, of which 50.8% (2,001 genes) were up-regulated and 49.2% (1,937 genes) down-regulated (Supplementary Fig. S12A and B). Most genes (454 up-regulated and 1267 down-regulated) were affected by *OsSGL* overexpression in young panicle of 3–6 cm in length before heading, and the fewest genes (803 up-regulated and 543 down-regulated) were affected in panicle over 15 cm 3 days before heading. More genes (1267) were down-regulated than up-regulated (454) in young panicle of 3–6 cm in length before heading, and in contrast, more genes (803 and 1008) were up-regulated than down-regulated (543 and 513) in panicles over 15 cm 3 days before heading and 10 days after heading. Only 80 DEGs overlapped among the samples (Supplementary Fig. S12A and B). Gene Ontology (GO) analysis revealed significant enrichments in genes associated with pathways related to stimulus response, cellular process, protein metabolism, hormone response, transcription and transport, and molecular functions including hydrolase, proteolysis, protein phosphorylation, DNA binding, ATP binding and transcription regulation (Supplementary Fig. S12C and D). As expected, hierarchical cluster analysis of associated GO-terms indicated that significantly regulated genes were mainly involved in abiotic-stress response, cytokinin (CK) signaling process, cell cycle, plant hormone response/biosynthesis/signaling, proteins of known molecular function and proteins with transcription factor binding activity (Fig. 4A–F).

**Figure 5:**
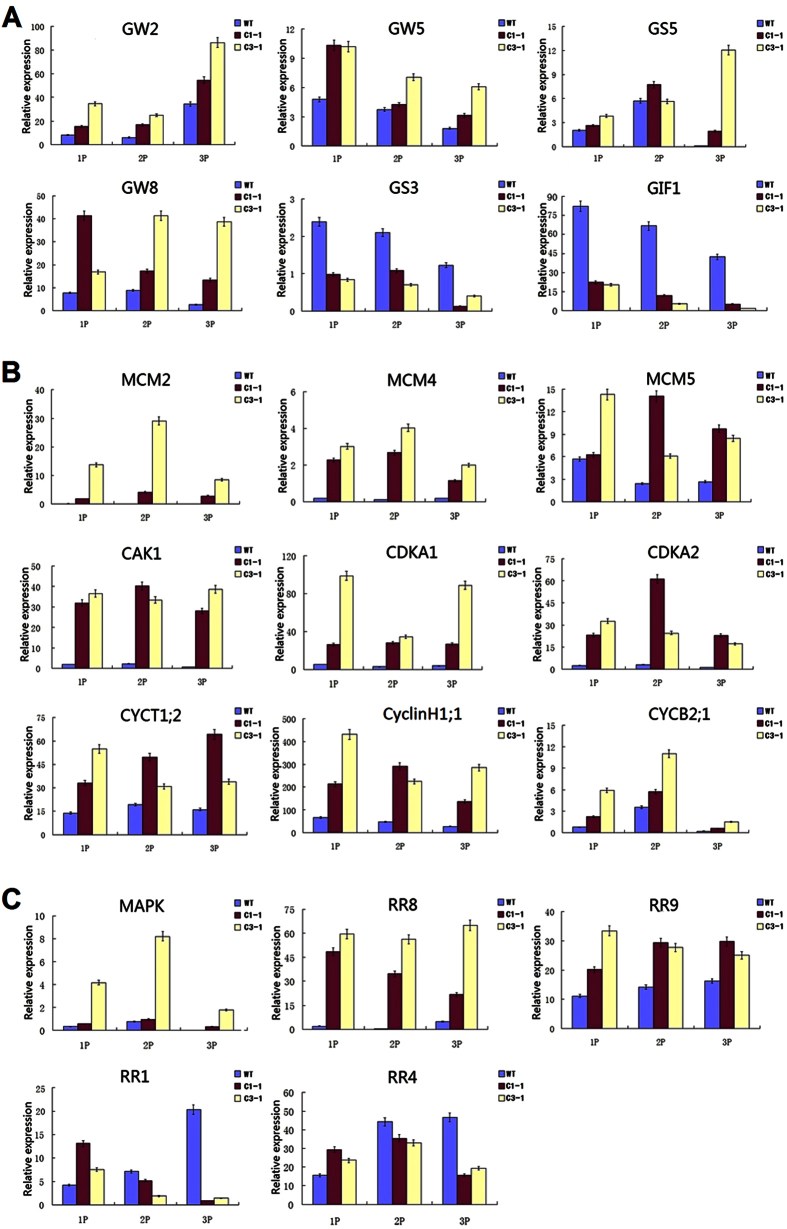
The relative expression levels of analyzed genes in two *OsSGL*-overexpressing transgenic lines and the wild-type. Genes were grouped by gene function and family. (**A**) Genes previously shown to regulate rice grain size. (**B**) Genes putatively associated with cell cycle G1/S-phase transitions. (**C**) Rice A-type Response Regulator gene (*OsRR*) family functioning in cytokinin signaling. Relative expression levels of each gene were determined by qRT-PCR with the RNA isolated from five panicles sampled, in three biological samples and three replicates. Values are measured as means ± s.e.m, and all *P* values are based in two-tailed *t*-tests. WT, wild type rice; C1–1 and C3–1, *OsSGL*-overexpressing transgenic rice lines; 1 P, young panicle of 3 to 6 cm in length before heading; 2 P, panicle over 15 cm 3 days before heading; 3 P, panicle 10 days after heading.

To confirm the microarray data and obtain further insight into the function of *OsSGL,* based on the functional annotation, we analyzed the expressions of 52 genes of interest by qRT-PCR in two 93-11-OE lines and the wild-type 93-11. The selected genes included 13 involved in grain shape, 4 in panicle architecture, 8 in CK signaling and 27 in cell cycle regulation (Supplementary Table S4)^26^,^27^,^28^,^29^,^30^. Compared with the wild-type, the transcript levels of four genes positively regulating grain size, *GW2, GW5, GS5* and *GW8*, were greatly elevated in the panicles of two 93-11-OE lines. In contrast, the expressions of two genes negatively regulating the grain size, *GS3* and *GIF1*, were significantly reduced (Fig. 5A). The expression levels of nine putative G1/S-phase genes *MCM2, MCM4, MCM5, CAK1, CDKA1, CDKA2, CYCT1;2, Cyclin1* and *CYCB2;1* were also elevated in the *OsSGL*-overexpressing lines (Fig. 5B). The expression levels of five CK signaling pathway-related genes, MAPK and four A-type *OsRR* (Response Regulator) genes (RR1, RR4, RR8 and RR9), were considerably changed in the transgenic plants (Fig. 5C). The other genes analyzed showed no significant differences in expression levels between the transgenic and wild-type samples. The correlation between the altered transcript levels of analyzed genes and *OsSGL* overexpression together with our finding that the multiple stress-responsive gene *OsSGL* contributes to abiotic stress tolerance, suggests that *OsSGL* may play a dual role in regulating stress response and meristematic activity.

## Discussion

*OsSGL* was identified and cloned as a novel pleiotropic stress-responsive gene from rice by using reverse genetics. Overexpression of *OsSGL* greatly and positively affected an array of agronomic traits in rice, including increases in grain length, grain number per panicle. This report provides an example for the isolation of yield-related genes through employing a different strategy, namely by selection based on their patterns of expression in plants under field conditions and verification by reverse genetics.

As sessile organisms, plants depend on a wide array of interconnected cellular stress response systems to protect them from environmental challenges. These cellular responses, helping in the prevention or alleviation of cellular damage, the re-establishment of homeostatic conditions and the resumption of growth, often mimics certain normal stages of development^29^,^30^. Thus, there is a broader overlapping set of genes and their cognate proteins involved in developmental processes and stress responses which are driven by systematic regulation of gene expression^31^,^32^,^33^,^34^. Our data showed that gene *OsSGL* participated in the regulation of both stress-tolerance and yield component traits in rice. Similarly, some rice genes, such as *MAPKs*^35^, *Hsp*^36^, *Orysa;EL2*^37^, *OsWRKY*^38^,^39^, *OsWOX*^40^, *OsMPS*^41^ and the wheat (*Triticum aestivum*) gene *dehythins (DHNs*)^42^,^43^ play various roles in both environmental stress defense and certain key development stages of plant tissues and organs.

Gene *OsSGL* contains no intron and encodes a small protein (255 aa) of previously unknown function that belongs to the DUF1645 protein family (Supplementary Fig. S1). Whether or not the intactness of the DUF1645 domain is essential for the effect of *OsSGL* on the increase of grain length of rice, and how it works is currently under investigation.

Alignment of the *OsSGL* nucleotide sequence and its ORF with homologs of other species showed a high DNA similarity (98%) only to common wild rice (*O. rufipogon*), but much lower DNA similarities to other species. Among the latter, the highest similarity detected (only 31%) was to a sorghum gene (XM_002453222.1), suggesting that gene *OsSGL* is quite specific to the rice genome. However, the polypeptide similarities of OsSGL to non-*Oryza* species were higher than those of DNA sequences, up to 53.3% with maize (NP_001145342.1), indicating that the function(s) of *OsSGL* may be more or less be conserved in plants closely related to *Oryza* species. The orthologs of *OsSGL* from maize, sorghum and wheat were cloned, and whether or not they may play a similar role to *OsSGL* in the increase of grain length is being analyzed by their hetero-expression in rice.

*OsSGL* is a powerful gene, and its overexpression not only significantly enhanced drought tolerance in rice, but also greatly and positively affected an array of traits, including large increases in grain length, grain weight and grain number per panicle and induced curling of the flag leaf, resulting in a significant increase in grain yield. Overexpression of *OsSGL* in rice resulted in an average increase of 24.8% in grain length, probably the largest increase of grain length so far caused by the change of a single gene activity. By comparison, loss-of-function mutations of three major negative regulators of grain length loci of rice, *GS3*^18^,^44^, *GL3.1/qGL3.1*^45^,^46^ and *TGW6*^21^, were reported to result in grain length increases of 10.5%, 10.8%, and 10.2%, respectively. Up-regulation of *GL7*^47^ and *GW7*^48^, two positive QTLs affecting rice grain length, were previously correlated to grain length increases of 7.8%, and 5.9%, respectively. Also, overexpression of the atypical bHLH protein genes, *PGL1*^49^ and *PGL2*^50^, were positively involved in rice grain length increases of 14.5% and 20.3%, respectively, by increasing cell length in the lemma/palea. Silencing of *APG*, the antagonist of *PGL1*, produced 13.0% longer grains in rice^49^. Overexpression of another gene, *BG2/GL3.2*, encoding a cytochrome P450 (*CYP78A13*) involved in brassinosteroids (BR) biosynthesis pathway, positively regulates grain size (20.8% longer) by mediating cell-cycle progression^51^.

The remarkable phenotypes of both long grain and curling flag leaf associated with overexpression of *OsSGL* would greatly facilitate the selection of positive transgenic plants and further downstream breeding. Overexpression of *BU1* (BR-induced rice gene), the closest homolog of *PGL2*, also caused 4.8% increase in grain length in addition to leaf bending and internode structure^52^. Overexpression of *CPB1/D11* (encoding a cytochrome P450 involved in BR biosynthesis pathway) not only influenced development of panicle architecture and plant height, but also had a larger leaf angle and greater grain length (19.1%)^53^. The various functional attributes of *OsSGL* make it a highly valuable and useful candidate gene for the study of substantial network of regulatory interactions and coordination of distinct pathways that underlie crop abiotic stresses and yield, and for crop improvement.

CKs, representing a group of phytohormones, regulate numerous biological processes, including cell proliferation^54^, reproductive development^55^, seed yield^56^ and responses to environmental stresses^57^, via a complex signaling network^58^. The intensive cross-talk between the different plant hormones results in synergetic or antagonistic interactions that play crucial roles in regulating every aspect of plant growth and development and the response of plants to biotic and abiotic stresses^59^,^60^,^61^. However, the identification of the players functioning downstream of the initial CK signal transduction pathway and how the CK response pathway integrates into the machinery regulating progression through the known cell cycle are only beginning to be appreciated^54^,^57^,^58^,^59^,^60^,^61^. To date, QTLs cloned for rice grain size and/or shape suggest that genes with various functions can positively or negatively affect grain yield, including genes that regulate cell proliferation and elongation through signaling pathways mediated by proteasomal degradation, phytohormones and G protein^62^,^63^. Our findings present the first evidence, to our knowledge, that the regulation of both stress-tolerance and yield component traits could be controlled through a positive modulator upstream of cytokinin signaling. Biotechnological strategies based on the modulation of CK levels have been examined with the aim of stabilizing agriculture yields^57^. It is possible that *OsSGL* may function as a positive modulator upstream of CK signaling or in an indirect manner affect the CK pathway, and that its overexpression may result in increases in stress-tolerance, in cell numbers by promoting mitotic division in the inflorescence meristem, and in cell size in plant tissues, ultimately resulting in increased seed size and production.

## Materials and Methods

### Plant material and stress treatments for microarray analysis

Seeds of rice cultivar PA64S (*O. sativa* L. ssp. *indica*), obtained from the Hunan Normal University (Changsha, Hunan, China), were sterilized with 70% ethanol for 1 min and then with 2% (w/v) sodium hypochlorite for 15 min, washed 3 times with sterile distilled water and soaked in water for 3 days at 25 °C (with daily water changes). Germinated seeds were transferred to an incubator with a photoperiod of 12 h light (30 °C)/12 h dark (25 °C) for 5 days, and then transplanted into pots (3 seedlings/pot) and grown under natural conditions prior to stress treatment. A temperature-controlled growth chamber was used for heat stress (45 °C, 2 h, under light) and cold stress (4 °C, 16 h, without light) treatments. For drought stress, plants were grown under natural conditions without watering for several days until 90% of the leaves coiled. After each treatment, leaves of 2 week-old seedlings, the first leaf below the flag leaf, and the corresponding panicles of rice plants at booting and heading stages were used to extract RNAs for microarray and qRT-PCR analyses. Young panicles of 5–8 cm long were harvested for samples at booting stage, and panicles after flowering 2–5 days were harvested for samples at heading stage.

### Microarray analysis

The Affymetrix GeneChip Rice Genome Array (Gene Expression Omnibus platform accession no. GPL2025) was employed for microarray hybridization. This array contains 51,279 probe sets that represent two rice cultivars, including 48,564 probe sets for *japonica* transcripts and 1,260 probe sets for *indica* transcripts. RNA samples were processed according to the instructions provided in the GeneChip^®^ Expression Analysis Technical Manual (2007 version) from Affymetrix. The arrays were washed and stained (streptavidin-phycoerythrin) in an Affymetrix Fluidics Station 4500 followed by scanning in a GeneChip Scanner 7 G using GeneChip Operating Software Version 1.5 as described in the supplier’s protocol. GeneChip Microarray analyses were performed according to the GeneChip Expression Analysis Technical Manual. The hybridization data were analyzed using GeneChip Operating Software (GCOS1.2) and dChip software (http://www.dchip.org). The expression levels of each gene were calculated from the signal intensities at each probe site after image scanning. A *P*-value adjustment was used in false discovery rate control for multiple testing. Significantly responding genes were selected by adjusted *P*-values less than 0.05. Genes that responded to at least one treatment were subjected to expression pattern analysis. Cluster analysis of those response genes under different stresses were performed using plots package.

### Quantitative Real-Time PCR analysis and validation

The total RNAs were used as templates with a one-step QuantiTect SYBR Green RT-PCR Kit (Qiagen), and qRT-PCR was performed using the gene-specific primers *OsSGL*-RT Forward and Reverse primers (Table S2) in an ABI 7900HT sequence detection system (Perkin-Elmer Applied Biosystems) according to the manufacturer’s instructions. The gene for 18 S *rRNA* was used as the internal control. Threshold cycles for each of the target genes and control were adjusted manually. Real-time PCR efficiencies (E) were calculated from the slopes of standard curves for each gene. Samples from the unstressed group were selected as calibrators. After normalization with the 18 S *rRNA* transcript, the data was analyzed using the comparative Ct method of Livak and Schmittgen^64^, and the relative *OsSGL* transcript levels in the various strains were averaged from three independent replicates of each samples.

### Vector construction and plant transformation

Relevant primers are listed in Supplementary Table S2, and various DNA fragments were amplified and introduced into different binary vectors for plant transformation. For *OsSGL* overexpression vector construction, the cDNA fragment with the whole open reading frame of *OsSGL* was amplified from total RNA of PA64S with *OsSGL* Forward and Reverse primers. And after verifying the sequence, the PCR fragment was inserted into the multi-cloning sites of the binary expression vector *pCAMBIA1300*. The resulting overexpression construct *pCaMV35S::OsSGL::NOS*, which carried *hpt*II (encoding hygromycin resistance) as a selection marker, was introduced into *A. tumefaciens* strain EH105 cells by electroporation. All the final constructs were sequenced to ensure the correctness of introduced segments and then were used to transform immature embryos of rice plants^65^. In total, we obtained 23 independent transgenic plants from 93-11, 28 from Huinong4, 58 from Minghui63, 67 from KH2 and 21 from TB309. All transgenic plants were grown under natural field conditions during the standard rice season in the experimental research field located at the Institute of Subtropical Agriculture, CAS, Changsha, Hunan, China. The planting density was 20 cm between plants in each row, and the rows were 20 cm apart, with one plant per hill. The area per plot was 13.34 m^2^. Field management, including irrigation, fertilizer application and pest control, followed the normal agricultural practice. The transcriptional levels of *OsSGL* were assessed using the middle part of the 7^th^ young leaf from different independent transgenic lines. Quantitative RT-PCR analysis showed that expression of *OsSGL* in all 93-11-OE transgenic lines was significantly enhanced compared to the level in wild type plants (Supplementary Fig. S3).

### Phenotypic measurements

Harvested rice grains were air-dried and stored at room temperature for at least two months before testing. Fully filled seeds (with hull) were used for measuring grain length, width and weight. In detail, thirty-six full seeds were randomly selected from each cultivar/line and divided into three groups equally. All seeds from each group were lined up length-wise along a Vernier caliper to measure seed length and then arranged breadthwise to measure seed width. Seed length and width were determined by averaging three measurements. Seed thickness was measured individually by using a Vernier caliper. More than 300 seeds of each plant were used to measure for one hundred-seed weight, which was then converted to 1,000-seed weight. For the measurement of panicle traits, three medium-sized main panicles were obtained from each transgenic and corresponding control plants. We measured the panicle length, the number of primary rachis branches, the number of secondary rachis branches, and the number of grains per panicle. For the measurement of the flag leaf traits, thirty healthy plants were randomly selected from each cultivar/line and divided into three groups equally. The flag leaf length (FLL, cm) and flag leaf width (FLW, cm) of first two panicles were measured, and the flag leaf area (FLA, cm^2^) was calculated using the formula: FLA = FLL × FLW × 0.75. Duncan or Dunnett tests were performed to compare the means of all traits for different allelic groups or cultivars/lines using SPSS 19.0 (SPSS Inc., IBM Company).

### Histological analysis and microscopy observation

Spikelet hulls at different developmental stages were harvested and fixed in a solution of 5% formaldehyde, 5% acetic acid and 60% ethanol for at least 24 h, dehydrated via an ethanol series, passed through freshly prepared 25%, 50%, 75% and 100% HistoResin over a 4-h period and finally embedded in HistoResin : HardenerThen (1:16) solution. Sections (3 μm) were cut using a rotary microtome, after staining with filtered 1% toluidine blue. All samples were microscopically examined (Leica DMR) and photographed. Area measurements of vascular elements were performed using the Leica Qwin software.

### Subcellular localization of the OsSGL protein in onion epidermal cells

To construct the vector for transient expression analysis, a 680 bp-long coding region of *OsSGL* were first amplified using the *OsSGL-*GFP Forward and Reverse primers. The intactness of the PCR fragment was verified by sequencing. It was then digested with restriction enzymes *Bam*H I and *Nco* I and ligated into the pJIT163-GFP (green fluorescence protein) intermediate vector, thus allowing the gene to be driven by the CaMV 35 S promoter. Next, the intermediate vector was digested with Kpn I and Xho I, and the fragment containing *pCaMV35S*::*OsSGL::GFP* was ligated into the pCAMBIA1300 vector. Vector *pCaMV35S::GFP* was used as a control. The constructs were delivered into onion epidermal cells by particle-bombardment as described previously^66^. At 24 h after bombardment, GFP fluorescence was observed with a Leica MZ16FA fluorescent stereomicroscope (Leica Microsystems).

### Histochemical localization of GUS activity in plants

For promoter-GUS fusion studies, a 1.2-kb genomic DNA fragment containing the 5′ promoter region of *OsSGL* gene was amplified by PCR based on the rice genome sequence information from DNA of PA64S. It was inserted into the upstream region of the GUS gene (*gusA, β-glucuronidase*) in pCAMBIA1301 (Cambia), resulting in a plasmid (*pOsSGL::gusA*) containing a GUS reporter gene driven by the *OsSGL* promoter. In total, we obtained 14 independent transgenic 93-11 plants carrying the *pOsSGL*::*gusA* construct. GUS activities were histochemically detected by using the method described by Jefferson^67^. Different organs of *pOsSGL*::*gusA* transgenic seedlings were incubated in a solution containing 50 mM NaPO_4_ buffer (pH 7.2), 5 mM K_3_Fe (CN)_6_, 5 mM K_4_Fe (CN)_6_, 0.1% Triton-100, and 1 mM X-Gluc, and incubated at 37 °C for 12 h. All samples were vacuum treated for 5 min before staining, and then the localization of GUS activity was performed in the transformants.

### Drought stress treatments of plants

A defined moderate drought resistance protocols with 20% (M/V) PEG6000 in hydroponics at two-leaf seedling stage was used. Seeds of WT and T_3_ transgenic plants were germinated on 1/2 MS medium at 25 °C for 3 days. Then 50 similar germinated seeds of transgenic plants from each transgenic line were planted in three rows (one plot) along with the WT control after a randomized complete block designation with three replicates. All plants were grown hydroponically in PVC (polyvinyl chloride) tanks (50 × 30 × 20 cm) filled with 10 L nutrient solution (pH 5.0–6.5). The nutrient solution consisted of NH_4_NO_3_ (1.43 mM), NaH_2_PO_4_.2H_2_O (0.37 mM), K_2_SO_4_ (0.5 mM), CaCl_2_ (1.00 mM) and MgSO_4_.7H_2_O (1.60 mM) and was changed every 3 days. Plants were grown for 7 days under a rain-out shelter with movable roofs in natural condition, air temperature ranging from 30 °C to 37 °C in the daytime and from 19 °C to 25 °C at night, and then 20% (M/V) PEG6000 was directly added into the solution. After the 7 day-long mild stress treatment (a condition that is not lethal to wild type plants), the lengths of roots and shoots of seedlings were measured.

## Additional Information

**Accession codes:** The GenBank accession number for *OsSGL* in spp. *japonica* cv. Nipponbare is AK108331.1.

**How to cite this article**: Wang, M. *et al*. OsGL, a novel pleiotropic stress-related gene enhances grain length and yield in rice. *Sci. Rep.* **6**, 38157; doi: 10.1038/srep38157 (2016).

**Publisher's note:** Springer Nature remains neutral with regard to jurisdictional claims in published maps and institutional affiliations.

## Supplementary Material

Supplementary Information
