# Supplementary material for: OsSGL, a novel pleiotropic stress-related gene enhances grain length and yield in rice
Source: Sci Rep. 2016 Dec 5;6:38157. doi: 10.1038/srep38157 (PMC5137154; doi:10.1038/srep38157)
Supplement: Supplementary Information [file srep38157-s1.doc]

**Supporting Online Material for**

***OsSGL*, a novel pleiotropic stress-related gene enhances**

**grain length and yield in rice**

Manling Wang, Xuedan Lu, Guoyun Xu, Xuming Yin, Yanchun Cui, Lifang Huang, Pedro S.C.F. Rocha and Xinjie Xia^1^

Key Laboratory of Agro-ecological Processes in Subtropical Region, Institute of Subtropical Agriculture, Chinese Academy of Sciences, Changsha, Hunan 410125, China

^1^To whom correspondence should be addressed. Email: jxxia@isa.ac.cn

**This file includes**

**Supplemental** **figures and figures legends**

**Supplemental tables**

**SUPPORTING INFORMATION**

**
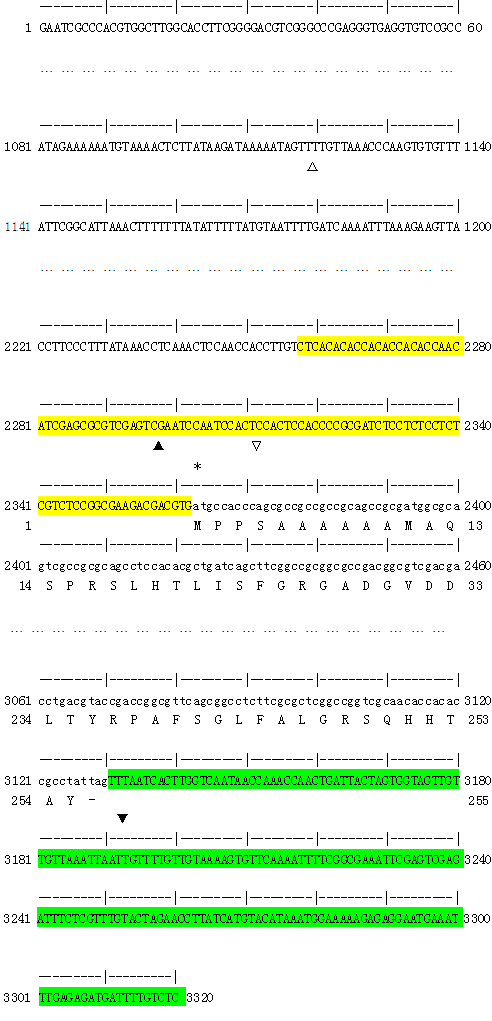
**

**Fig. S1.** The DNA and corresponding deduced amino acids sequences of gene *OsSGL.* The nucleotide and amino acid positions are indicated on the left and right; △ and ▽, the beginning and the end of the promoter region cloned for the GUS analysis, respectively; ▲ and ▼, the beginning and the end of the *OsSGL* sequence cloned for the overexpression construct, respectively; *, presumed translational start codon; —, translational stop codon; 5’UTR, shadowed in yellow; 3’UTR, shadowed in green.


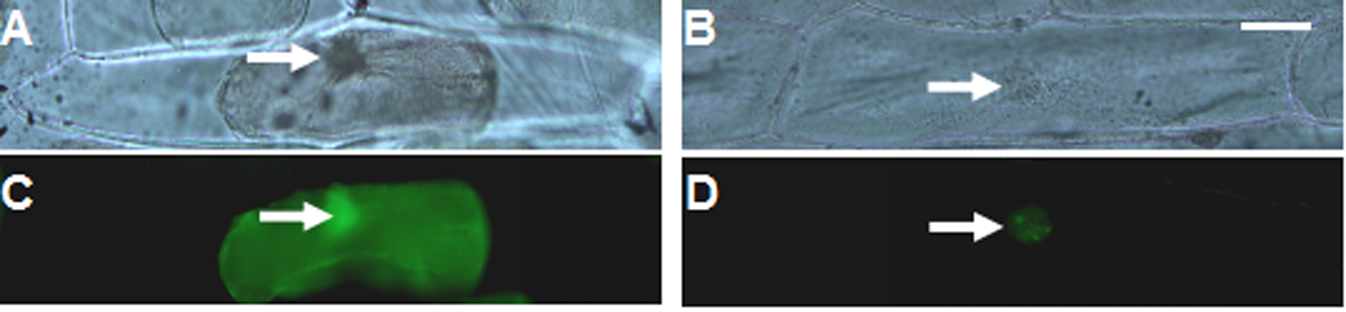


**Fig. S2.** The subcellular localization of *OsSGL* protein in onion epidermis cells. ***(A)***, ***(C)*** Microscopy-based localization of the GFP control (35S::GFP vector); ***(B)***, ***(D)*** The OsSGL-GFP fusion protein is detected within the nucleus. The upper panel represents bright field images; the lower panel shows green fluorescence signal of the same image fields detected at 488 nm. The arrows indicate the cell nuclei. Scale bar, 50 µm.


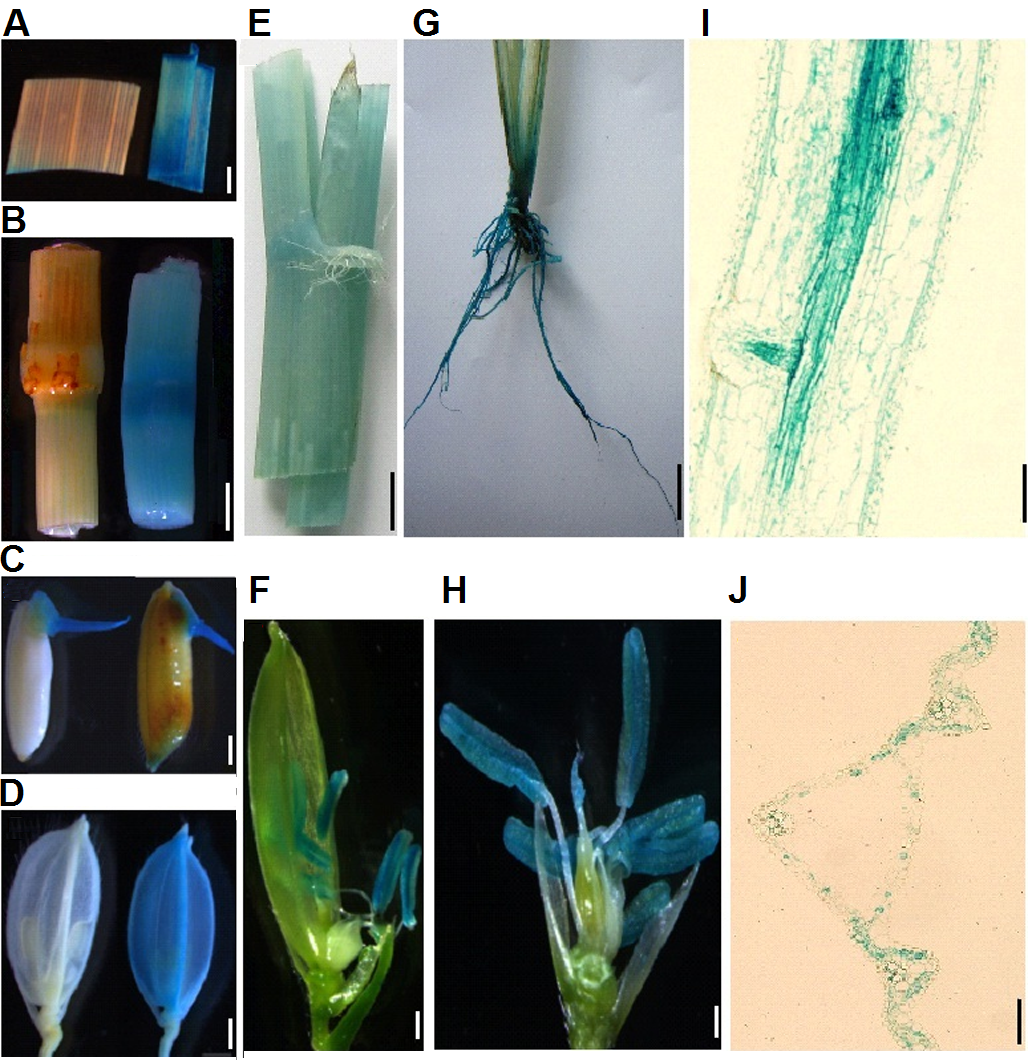


**Fig. S3.** The expression pattern of gene *OsSGL* in transgenic rice plants, as revealed by using the *OsSGL* promoter-GUS fusion construct. GUS expressions detected in leaf *(A)*, internode *(B)*, coleoptile *(C)*, leaf sheath *(E)*, root *(G)*, hulls of young spikelets *(D)*, stamen *(F)* and pistil *(H)* of mature spikelets before flowering. Scale bar, 1 cm for *A*, *B*, *E*, *G*; 20 mm for *C*, *D*, *F* and *H*. *(I)* longitudinal section of rice root at seedling stage AT . Red arrow indicates lateral/fibrous root apical meristem. *(J)* Transverse section of leaf blade; Black arrow indicates large and small vascular bundles. Scale bar, 50 μm.


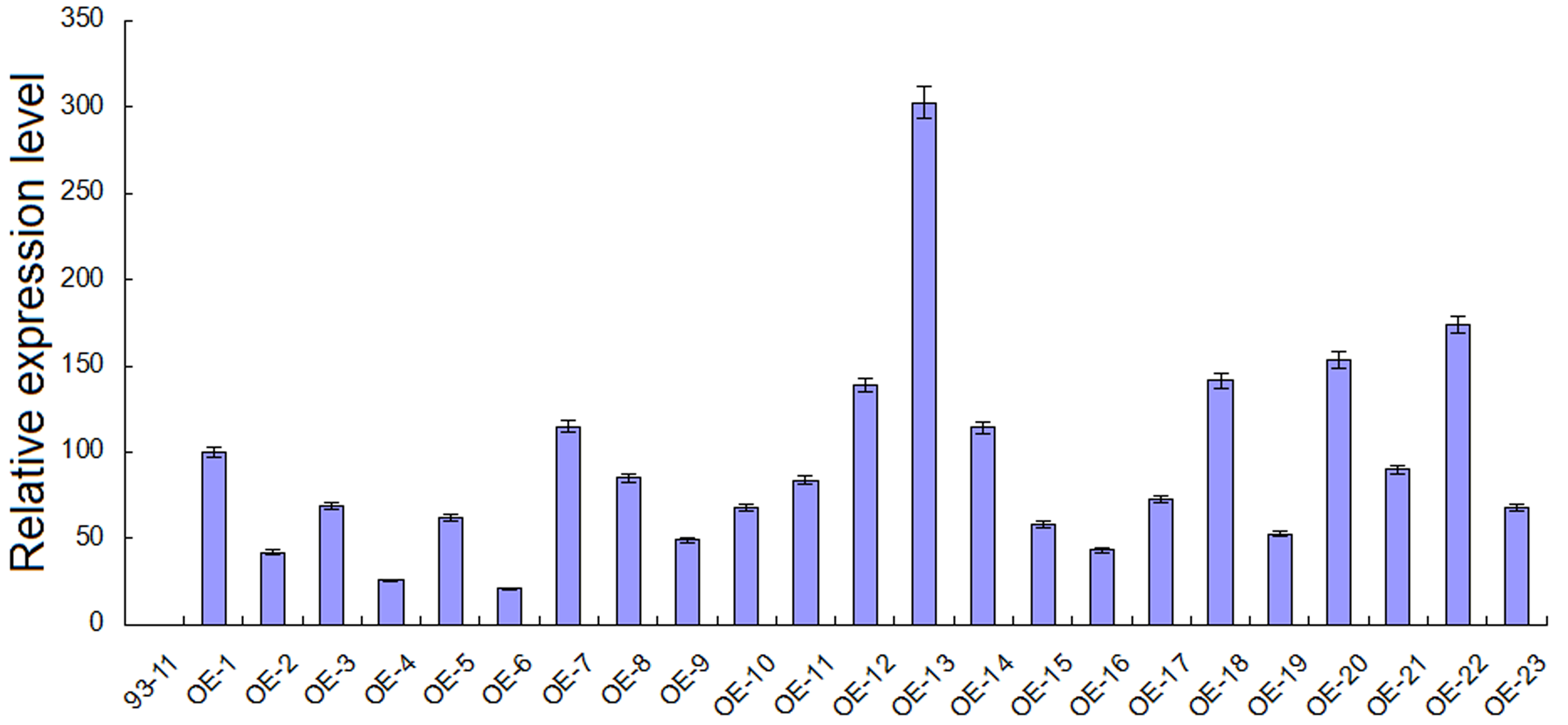


**Fig. S4.** The expression levels of *OsSGL* in 93-11-OE transgenic rice lines detected by quantitative Real-Time PCR using primers *OsSGL*-RT. 93-11, wild-type; OE-1 – OE-23, T_0_ transgenic lines. All data are based on three biological replicates. Error bars, s.e.m.

**
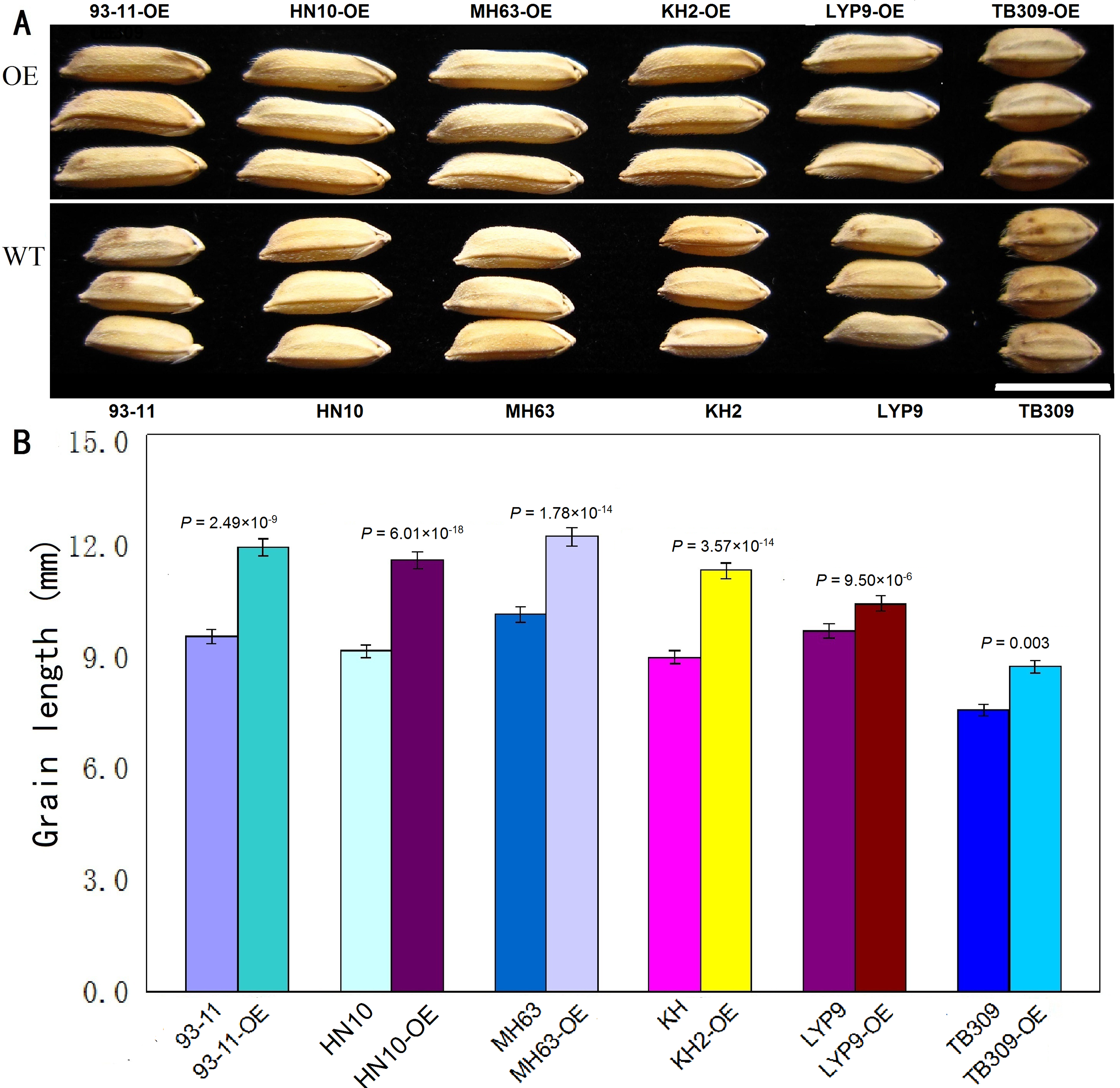
**

**Fig. S5.** Grains of the T1 generation of transgenic plants compared with the corresponding wild-type variety. (***A***) Gross morphology of rice grains between wild-type and *OsSGL*-overexpression transgenic lines. Scale bar, 1 cm. (***B***) Comparisons of grain length. Data are given as mean ± s.e.m. Student’s tests were used to generate the *P* values. WT, wild-type; OE, overexpression transgenic lines; HN10, Huinong 10; MH63, Minghui 63; LYP9, Liangyoupei 9; TB309, Taibei309. 93-11, HN10, MH63, KH2, LYP9, TB309, wild type; 93-11-OE, HN10-OE, MH63-OE, KH2-OE, LYP9-OE, TB309-OE, corresponding overexpression transgenic lines.


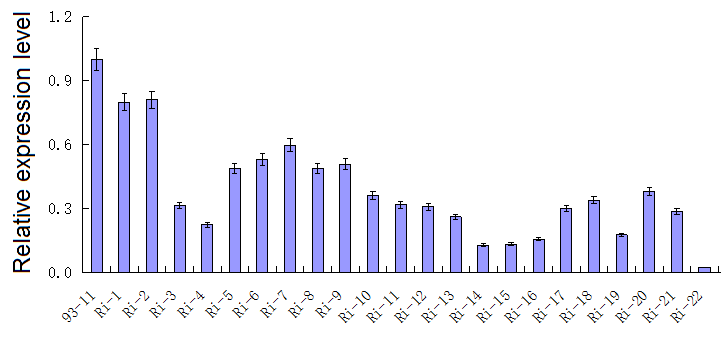


**Fig. S6.** The expression levels of *OsSGL* in 93-11-RNAi transgenic rice lines detected by quantitative Real-Time PCR using primers *OsSGL*-RT. 93-11, wild-type; Ri-1 – Ri-22, T_0_ transgenic lines. All data are based on three biological replicates. Error bars, s.e.m.


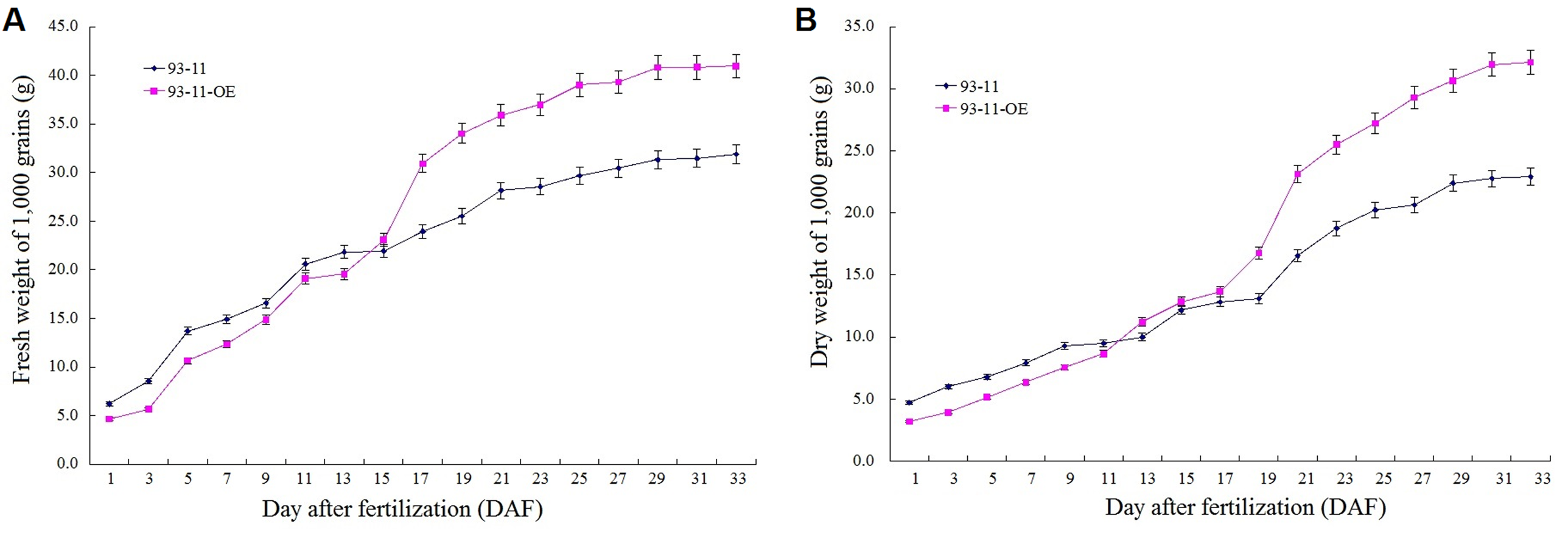


**Fig. S7.** Characterization of grain milk filling in 93-11 and 93-11-OE revealed the time course of the fresh weight ***(A)*** and dry weight ***(B)*** increase of brown grains. Data are the means ±SD (n = 40 plants).


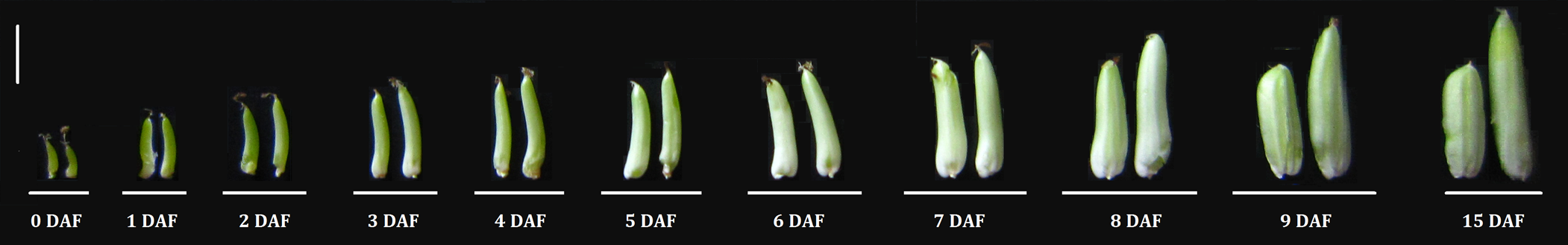


**Fig. S8.** Comparisons of rice ovaries between 93-11(left) and 93-11-OE (right) at indicated days after fertilization (DAF). Scale bar, 5 mm.


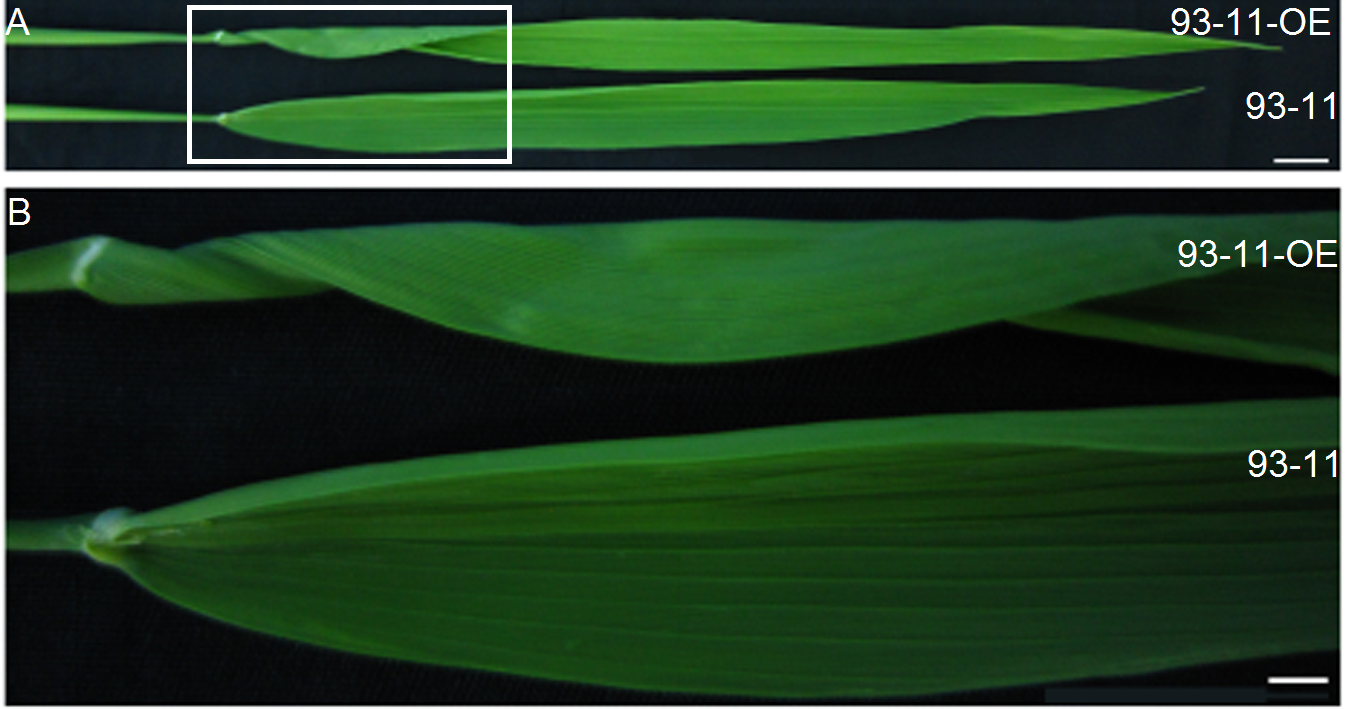


**Fig. S9.** Flag leaf morphology of 93-11 and 93-11-OE transgenic lines at heading stage. (***A***) 93-11-OE leaf spiraled slightly at the base of the leaf blade, whereas wild type 93-11 leaves were flat. (B) Magnifications of indicated squares cut at the coiled base of flag leaf. 93-11, wild type; 93-11-OE, transgenic overexpression lines. Scale bar, 2 cm, 0.5 cm.


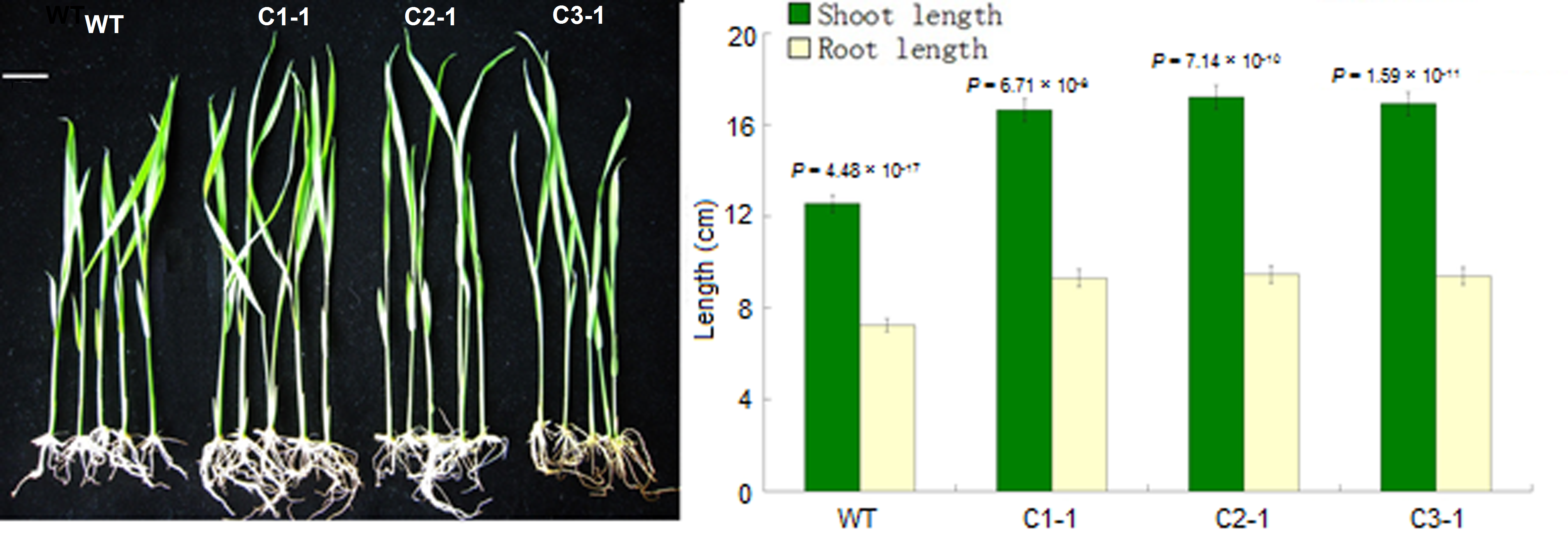


**Fig. S10.** Seedlings of 93-11 and three 93-11-OE transgenic lines under normal growth condition for 11 days. WT, wild-type 93-11; C1-1, C2-1, C3-1, transgenic lines; Scale bar, 1cm. Data are given as mean ± s.e.m. Student’s tests were used to generate the *P* values.


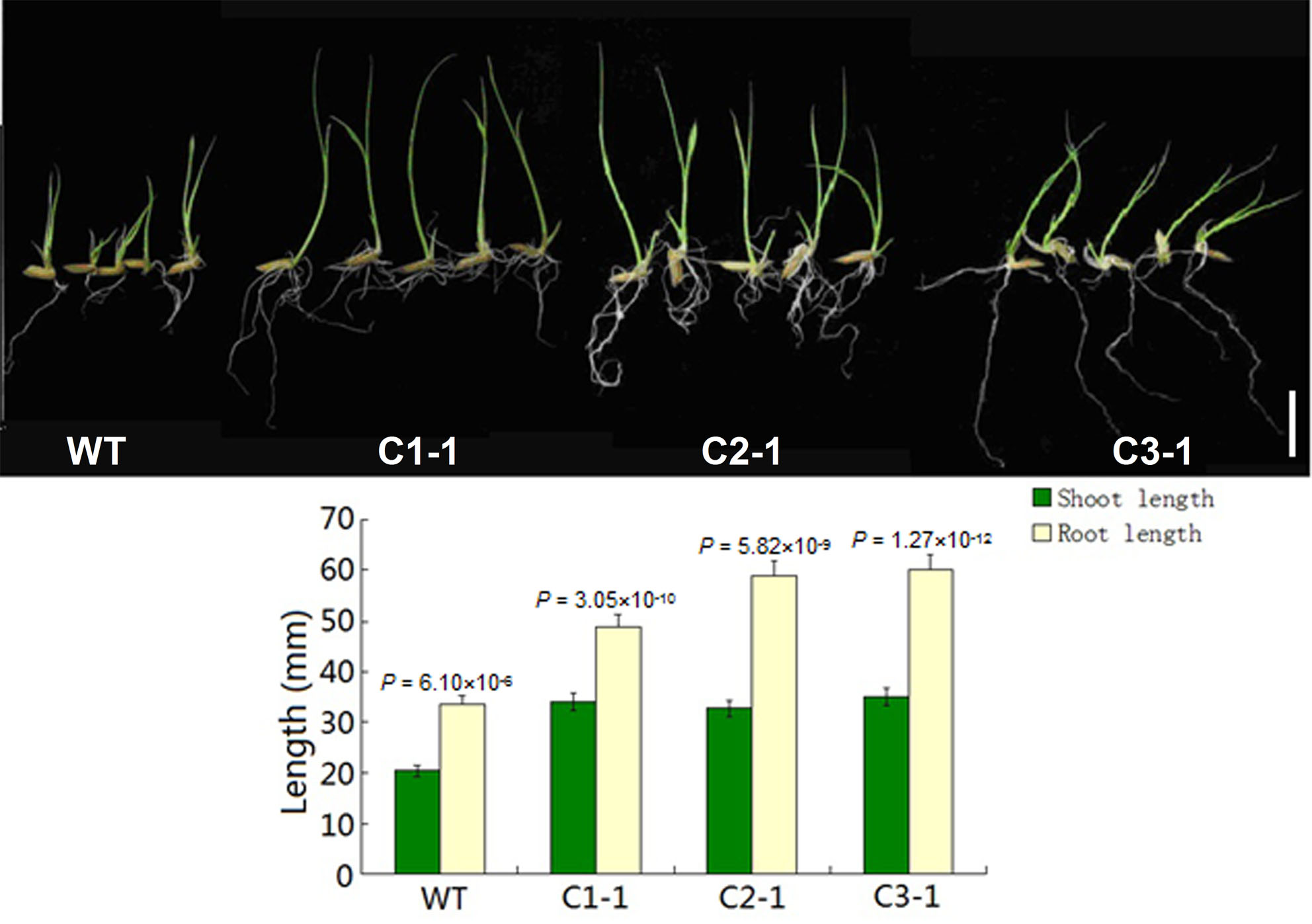


**Fig. S11.** 10 days-old seedlings of 93-11 and three 93-11-OE transgenic lines treated with 20% (M/V) PEG6000 for one week as moderate drought stress treatment. WT, wild-type 93-11; C1-1, C2-1, C3-1, transgenic lines; Scale bar, 1cm. Data are given as mean ± s.e.m. Student’s tests were used to generate the *P* values.


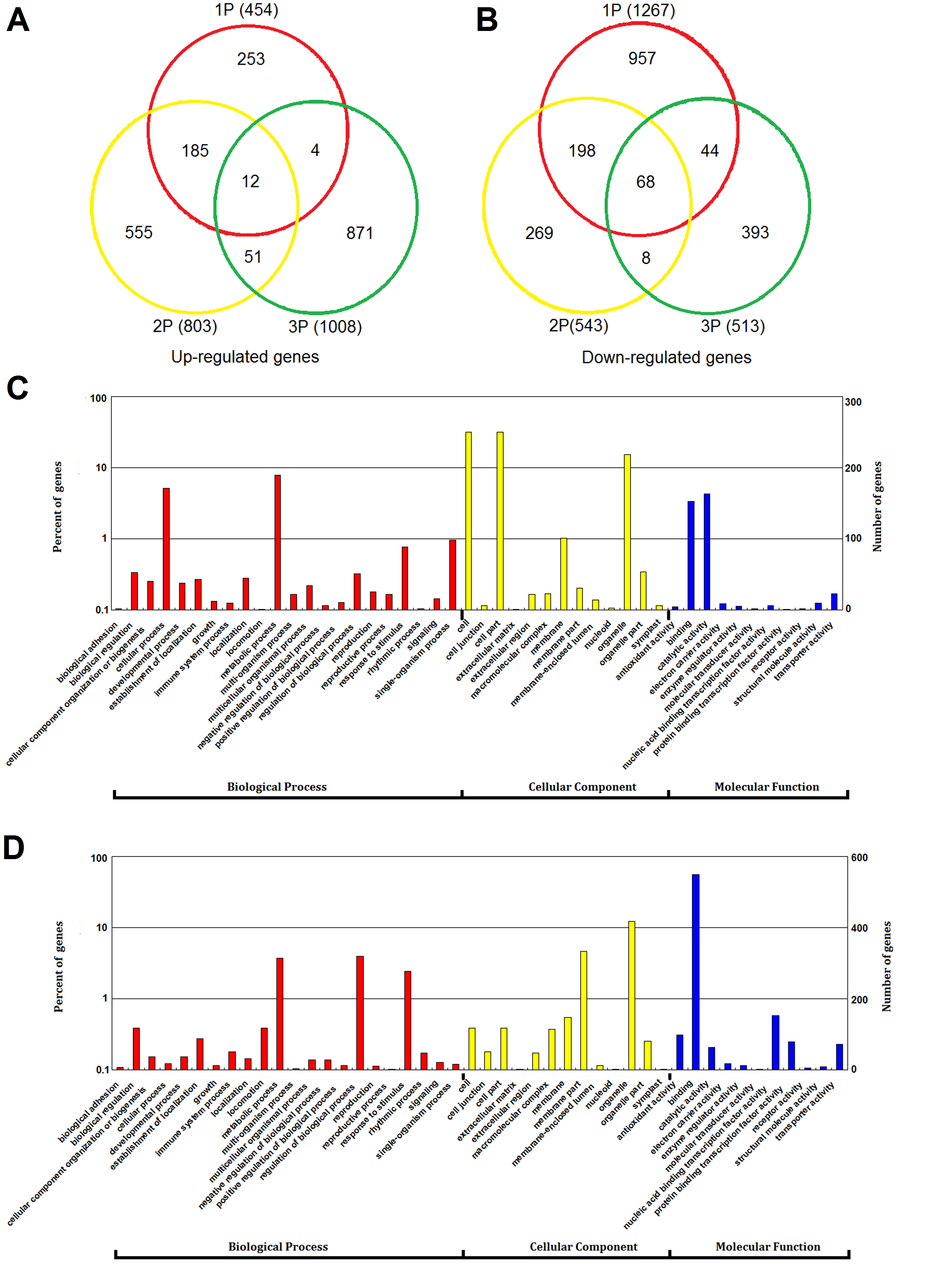


**Fig. S12.** Microarray analysis showed that the overexpression of *OsSGL* in transgenic plants altered transcriptions of a wide variety of genes involved in different biological processes and molecular functions. Venn diagrams show the unique and overlapped gene numbers of the selected up- (***A***) and down-regulated genes (***B***). Significantly enriched GO-terms show representative biological processes, cellular components and molecular function categories of up- (***C***) and down-regulated genes (***D***) identified in ***A*** and ***B***, respectively. 1P, young panicle of 3 to 6 cm in length before heading; 2P, panicle over 15 cm 3 days before heading; 3P, panicle 10 days after heading.

**Table S1 Changes of *OsSGL* expression levels under different stresses (up-regulated fold changes relative to the controls).**

| Organs at different development stages | | Methods | | | | | |
| --- | --- | --- | --- | --- | --- | --- | --- |
|  |  | Microarray | | | qReal-Time PCR | | |
|  |  | Cold | Drought | Heat | Cold | Drought | Heat |
|  | Leaf / Seedling | 33.86 | — | — | 7.23 | 1.13 | 2.28 |
|  | Leaf / Booting | 13.16 | 1.11 | 3.54 | 63.57 | 8.04 | 3.35 |
|  | Panicle / Booting, | 27.00 | 7.03 | — | 2.90 | 2.40 | 1.31 |
|  | Leaf / Heading | — | — | 4.67 | — | — | — |
|  | Panicle / Heading | 2.61 | 3.59 | — | — | — | — |

Response expression levels were measured as log_2_ fold changes. All qRT-PCR data are based on three biological replicates. —, data are unavailable.

**Table S2 PCR primers used for cloning and functional analysis of *OsSGL***

| Name | Forward primer(5’-3’) | Reverse primer(5’-3’) |
| --- | --- | --- |
| *OsSGL*-RT | CCGCCATCATCCAAACTGA | GGTGACCACGCCCTTCTTC |
| *OsSGL* | GGATCCCTAAGAATCCAATCCACTCCACTCCACC | CCCGGGCTAATAGGCGGTGTGGTGTTGCG |
| *OsSGL-*GFP | GGATCCCTAAGAATCCAATCCACTCCACTC | CCATGGCGTTATAGGCGGTGTGGTGTTGC |
| *OsSGL*-GUS | TTGTGGATCCCAAGTGTGTTTATTCGG | AGTGCCATGGATTCGACTCGACG |
| *OsSGL-*RNAi-1 | AAGCTTCGACAGGTTCCCCAAGAG | GATATCATAGGCGGTGTGGTGTTG |
| *OsSGL*-RNAi-2 | GAGCTCCGACAGGTTCCCCAAGAG | GGATCCATAGGCGGTGTGGTGTTG |
| 18S | CGTCCCTGCCCTTTGTACAC | CGAACACTTCACCGGATCATT |
| *hpt* II | CGCAAGGAATCGGTCAATACAC | AAATTGCCGTCAACCAAGCTC |

**Table S3 Phenotypical measurements (mean±s.e.m) of two cultivars 93-11 and TB309 and their corresponding T1 transgenic plants (OE).**

| Traits | 93-11 | 93-11-OE | *P-values* | TB309 | TB309-OE | *P-values* |
| --- | --- | --- | --- | --- | --- | --- |
| Number of panicles per plants | 12.8±1.280 | 17.4±0.839 | 2.599E-07 | 14.9±1.469 | 25.6±1.542 | 3.74E-05 |
| Main panicle length (cm) | 22.54±0.289 | 27.55±0.395 | 0.00011 | 18.14±0.417 | 22.81±0.436 | 0.005 |
| Number of spikelets per panicle | 168.8±6.598 | 206.3±1.448 | 2.63E-07 | 115.2±3.835 | 144.4±7.295 | 1.32E-05 |
| Number of grains per panicles | 149.1±5.998 | 187.4±1.022 | 1.232E-06 | 104.2±3.886 | 137.9±6.551 | *0.942* |
| Seed setting (%) | 88.33 | 90.84 |  | 90.45 | 95.52 |  |
| Grain length (mm) | 9.58±0.437 | 11.96±0.589 | 2.485E-09 | 7.60±0.053 | 8.75±0.070 | 0.0030 |
| Grain width (mm) | 2.91±0.015 | 2.66±0.023 | 1.284E-05 | 3.96±0.045 | 3.67±0.054 | 0.0003 |
| Grain length-to-width ratio | 3.29 | 4.42 |  | 2.18 | 2.38 |  |
| 1,000-grain weight (g) | 32.10±0.264 | 37.34±0.168 | 4.479E-08 | 28.07±0.632 | 31.74±0.785 | 0.0030 |
| Flag leaf length (cm) | 27.09±0.773 | 37.92±1.203 | 4.32E-05 | 34.98±1.123 | 37.63±0.842 | *0.236* |
| Flag leaf width (cm) | 2.03±0.023 | 1.77±0.029 | 8.68E-05 | 1.82±0.034 | 1.59±0.031 | 0.0007 |
| Plant height (cm to panicle) | 125.10±1.167 | 110.19±0.778 | 2.6E-07 | 109.0±1.862 | 102.08±2.010 | 4.76E-05 |
| Days to 50% flowering | 110 | 117 |  | 90 | 96 |  |
| Estimated days from seeding to ripen | 156 | 163 |  | 120 | 126 |  |

All data presented were from rice plants planted in randomized complete block design with three replicates under natural condition in Changsha, China in 2012. OE, transgenic plants harboring the *OsSGL*-overexpressing construct with the coding sequence of *OsSGL* driven by the CaMV35S promoter. *P*-values produced by two-tailed t-test, *P*-values higher than 0.05 are shown in italics.

**Table S4 Primer pairs used for qRT-PCR analysis of the effect of *OsSGL* on the expression of 52selected genes**

| Gene | GenBank ID | Affy NO. | Forward primer (5’-3’) | Reverse primer (5’-3’) |
| --- | --- | --- | --- | --- |
| *GW2* | EF447275.1 |  | CTGCAGCAGGGAAGTGGT | ATGTCTGAGCCCGCGTAG |
| *GW5* | CL971152.1 |  | ACCACTCCGTCCTCCTCCGCTT | AGGAGGACTAACGGGCGCTG |
| *Gnla* | AB205193.1 |  | GATAGCCTACAAGCAGTA | GCCTTTGGATCATACTTG |
| *DEP1* | FJ039905.1 |  | CCGTTTCTCGTTCTGGAT | ATCTGTGCCTCCTTCTCT |
| *Ghd7* | EU286800.1 |  | AGGTGCTACGAGAAGCAAATCC | GGGCCTCATCTCGGCATAG |
| *GIF1* | EU095553.1 |  | CATCGCGCAACCCGAACATG | TGTCGATCAGGCTCCTCAGAG |
| *OsSPL14* | AK107191.1 | Os.6636.1.S1_at | CAGCTACCACATGAAGTC | CTACAGAGACCAATCCATC |
| *GS3* | DQ355996.1 |  | CATCGGAGAAGCGAAGTCA | CAGCAGCAGATCCAGGAGA |
| *GW8/OsSPL16* | AK109469.1 | Os.56157.1.A1_at | AGGAGTTTGATGAGGCCAAG | GCGTGTAGTATGGGCTCTCC |
| *GS5* | JN256055.1 |  | AGTGGACTGCTTCCAGGGAAG | CACGCAGTACCGAGAACTGA |
| *FZP* | AK105365.1 | Os.32291.1.S1_at | CTCCAGCATGTCGTCGTC | CACCACGCTGCTCAGGTA |
| *LAX1* | AB115668.1 | Os.38423.1.S1_at | GATGACGACGCTGGAGAT | GACATTGCACACCGAGTAG |
| *LAX2* | AB669025.1 |  | GCCATCCACTACGTCAAGT | TGGACGAAGACACAGCAAGG |
| *RCN1* | AF159882.1 | Os.49726.1.S1_at | GACCTGCGATCTTTCTTCAC | GACAATTGGAGCTGCATTTC |
| *GL3.1* | AK288069.1 |  | TCACAACTCCCAGGATAGG | TTTGTCTCGCTCGCTCAT |
| *qSW5* | AB433345.1 |  | TATGCCGCTCCGTCCTCCTC | TGCAGCTCCTCCTGCCATC |
| SRS3 | AK064328.1 | Os.8608.1.S1_a_at | AGAGGTGAACTTGGAGCTG | GATGAGGATGATGATGACAT |
| *OsCLV1/FON1* | AB182388.1 |  | ACATGCTCTCCAACCCAAACTCG | TAGCCGCTAATTAAGCGCACAC |
| *FOS1* | AB455109.1 |  | CGAACAACCGAATATCTG | GAACGAGAACATCCAATC |
| *OsCLV3/FON2* | AB245090.1 |  | AGACGAATGGCTAGTATT | CTCTCATCCGATTCCTAC |
| *CYCD3;1* | AK103499.1 | Os.47738.1.S1_at | CCTTCCACACTGACGGTACAGTT | TGCCGCTGCCAAATAGACA |
| *CYCD4;1* | AK063940.1 | Os.18607.1.S1_at | GCCATGGAGTTGATACATCCAA | CCAGTAGGGCTCCGTGGAAT |
| *CAK1A* | AK120969.1 | Os.11911.1.S1_at | GACCGACAAGGGTTTCAGCAT | CCAGCATGTTCAGGAAGATACAAT |
| *CDKA1* | AK062097.1 | Os.11723.3.S1_at | GGTTTGGACCTTCTCTCTAAAATGC | AGAGCCTGTCTAGCTGTGATCCTT |
| *CDKA2* | AP004885.1 |  | CGAGATTTGAAGCCCCAGAA | TCCGCGAGCTTCAATGAGTT |
| *CYCT1;2* | AK107537.1 | Os.17669.1.S1_a_at | GCATTTGTTGCAGCTCAAG | TCACCACTTCGCTGACTTATTG |
| *CYCT1;3* | AK120409.1 | Os.11817.1.S1_a_at | TGGAGAAACGGAGGAAAT | GAACGGTACTCTTGGCTGT |
| *OsE2F2* | AK059233.1 | Os.424.1.S1_at | TGTTGGTGGCTGCCGATAT | CGCCAGGTGCACCCTTT |
| *OsE2F3* | AK067581.1 | Os.52509.1.S1_at | CATCCACTCCTGCCAGTTCT | CACGAGGTCCTTTTATTGCGAT |
| *OsE2F1* | AB041725.1 | Os.423.1.S1_a_at | ATGGGTCCTGTTGATTGCT | GGATGGCTCTGATGTATGCT |
| *CycH1;1* | AK105468.1 | Os.3905.1.S1_at | GCAAGGCACCTGCAGCTT | AGGCAGCCTTTGTACAGATCCT |
| *MCM2* | XM_015759702.1 |  | AAGTTGGCAAAAGATCCACGG | CCCCCAAACATAGCTAGTGCAA |
| *MCM3* | XP_015637328.1 |  | TTCATGCGTCACTAAATGCGAG | TGAATCTGGAAGCCCAATGTTC |
| *MCM4* | XM_015774693.1 |  | CCCGAATGCGATTCTCTGAA | ACCAGTGGCATGATCAGTTGC |
| *MCM5* | KM823080.1 |  | AAGGAGAACTGCCTGTCCATGA | AGTGGCCTTAGCTTTCACCCTC |
| *CYCP4;1* | AK107529.1 | Os.46852.1.S1_at | AGATGGTCGCCGGCTTCT | ATGGAGAGTAGGAGTAGGAG |
| *CDT2* | AL954854.3 |  | AACCGCACCAAACACTGGAA | GCAATTCACCATCTGCACTGG |
| *CYCA2;1* | AK106653.1 | Os.9590.1.S1_at | AGGTTGTCAAGATGGAGAGCGA | CGCTTTTTGTCTTCCTGGCA |
| *CYCA1;1* | AB024986.1 | Os.4195.1.S1_at | GTTTCGGTTGACGAGACGATGT | CGCTGCAAGGAACCTAGAACTG |
| *CYCB2.1* | AB024987.1 | Os.9246.1.S1_at | AAGTTTGGCCAGGAGTGAGCA | TCAAGAGCATCAGCGTCGAGA |
| *CYCB2.2* | AK070518.1 | Os.2693.1.S1_x_at | CTCAAGGCTGCACAATCTGACA | GCATTGACGGCTGGAATTTG |
| *CYCB1;1* | AK111939.1 | Os.11347.1.S1_at | CACTCTCAAGCACCACACTGGA | ACAACCCTCAGCTTGCTCTCAG |
| *CDKB* | AK242651.1 |  | AAGTTTGGCCAGGAGTGAGCA | TCAAGAGCATCAGCGTCGAGA |
| *KRP1* | AK103084.1 | Os.19953.1.S1_at | CCGCGAGAGGAGAGAAACAA | CGACACACTGCTAAACTGAAC |
| *MAPK6* | AB183398.1 | OsAffx.29964.2.S1_x_at | ACAGAGCAGCCGAATTTTGAGA | TTCAGCGAAGCTCACACTTGG |
| *OsRR1* | AK072736.1 | Os.8117.1.S1_at | AGGATCAGCAGATGCATGAATG | GAGACGCTGTACGTCCTTGCTT |
| *OsRR2* | AK070645.1 | Os.23942.1.A1_at | ACGATCTTCTCAAAGCCATCAAG | TGAGAGGCTTAAGGATGAAATCCT |
| *OsRR4* | AK101721.1 | Os.15908.1.S1_s_at | TCTTCTGAGAATGTGCCTGCAA | GCTTGACAGGTTTCAGGAAGAACT |
| *OsRR6* | AK059734.1 | Os.24952.1.S1_at | GTCCCCAACGTCAACATGATC | CACGTTCTCCGACGACATGAT |
| *OsRR7* | AJ938076.1 |  | TGCTCAAGAAGATCAAGGAATCG | GGCACGTTCTCTGACGACATTAT |
| *OsRR8* | BR000317.1 |  | CCA GAC ATG ACC GGC TAT AAC C | AAG CAA TTA CAA CCG GGA GAT G |
| *OsRR9* | AJ938078.1 |  | TCATGAGGACAGCCCAATTTCTA | TGCAGTAGTCTGTGATGATCAGGTT |
